# In vitro assessment of cytokine modulation by choline salicylate, hyaluronic acid, lidocaine-based six different teething gels in human gingival fibroblast cells

**DOI:** 10.1007/s10266-025-01120-6

**Published:** 2025-05-13

**Authors:** Melike Tiras, Burcu Gucyetmez Topal, Sefa Celik, Serkan Sen

**Affiliations:** 1 Sivas Dental Health Hospital, Sivas, Turkey; 2 Faculty of Dentistry, Department of Paediatric Dentistry, Afyonkarahisar Health Sciences University, Afyonkarahisar, Turkey; 3 Faculty of Medicine, Department of Medical Biochemistry, Afyonkarahisar Health Sciences University, Afyonkarahisar, Turkey; 4 Department of Medical Laboratory Techniques, Ataturk Vocational School of Health Services, Afyonkarahisar Health Sciences University, Afyonkarahisar, Turkey

**Keywords:** Dentistry, Gingiva, Inflammation, Interleukins

## Abstract

Teething gels are used to treat various oral mucosal conditions and inflammation. The effect of teething gels on cytokines involved in the inflammatory process is unknown. This study aimed to assess the influence of teething gels on the levels of inflammatory cytokines in human gingival fibroblasts using an in vitro assay. In this study, MTT (3-[4,5-methylthiazol-2-yl]-2,5-diphenyl-tetrazolium bromide) test was used to calculate the values that provide 50% survival of the cells with different teething gels applied to human gingival fibroblast cells. Using Enzyme-Linked Immunosorbent Assay kits, Interleukin-1β, Interleukin-6, Interleukin-8, Tumor necrosis factor-α, and Interleukin-10 cytokine levels were measured in the control group and in fibroblast cells treated with each teething gel. The statistical analysis of the data was done with SPSS program. All of the teething gels caused various levels of suppression of the cytokines governing the inflammatory process (p < 0.05). Interleukin-1β levels were significantly lower in the hyaluronic acid group than in the lidocaine group (p < 0.05). Interleukin-8 levels were significantly lower in all teething gel groups compared with the control group (p < 0.0001). In conclusion, all of the teething gels in this study were able to suppress the cytokines that regulate the inflammatory process. Considering the cytotoxic side effects of other teething gels, it is concluded that hyaluronic acid-based teething gels have a safe and tolerable profile in the suppression of cytokines that manage the inflammation process in symptom-directed treatments.

**Graphical abstract:**

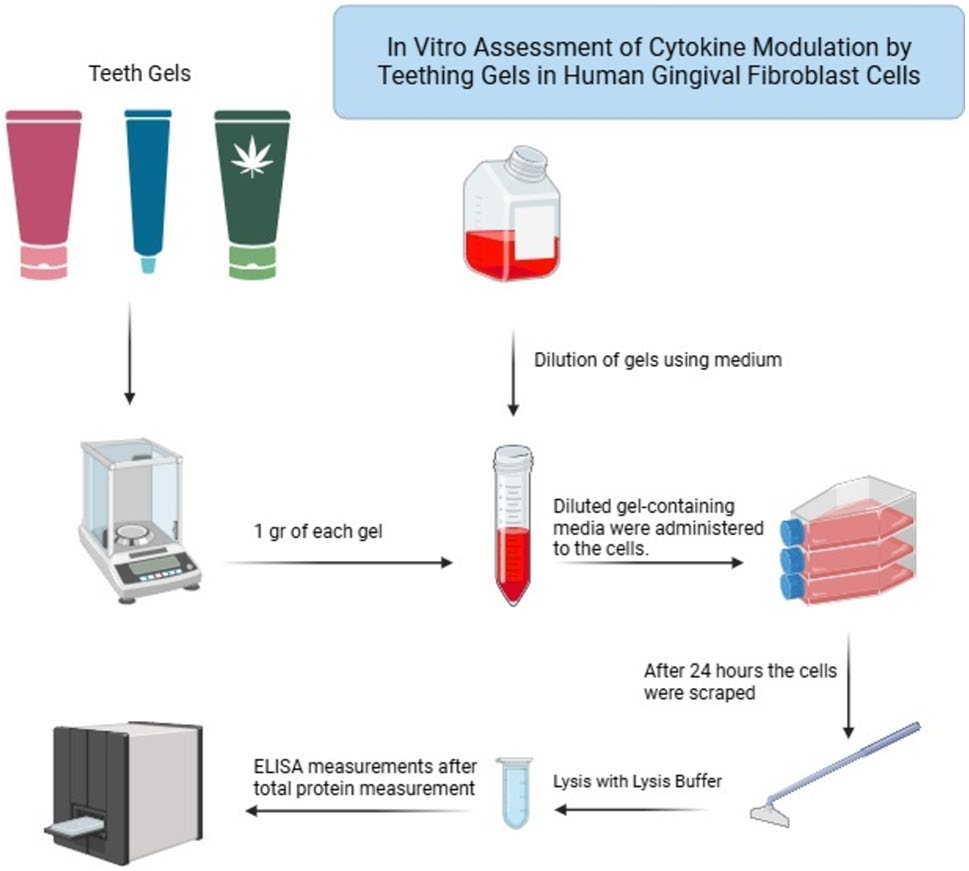

## Introduction

Teething gels are used in various oral mucosal conditions, including alleviating pain in intraoral lesions, expediting healing, addressing periodontal issues, and managing gingival inflammation linked to infant teething. Teething gels are favored owing to their high-water content, minimal mucosal irritation, ease of application, direct mucosal impact, and rapid dispersal [1, 2].

Teething gels are commonly employed among different age groups, especially for infants younger than 6 months old; they are the second most common medication after paracetamol-derived medications [2]. Commercial teething gels have diverse formulations with various active components, including hyaluronic acid, lidocaine hydrochloride, and choline salicylate. These elements, which have analgesic, anesthetic, and anti-inflammatory properties, aim to relieve local oral and gingival symptoms. However, data regarding the safety and efficacy of such childhood medications are scarce [3]. In the available literature, there are survey studies on the preference of teething gels by parents for their children and their recommendation by health professionals, case reports on their cytotoxicity, in-vitro studies on human gingival fibroblasts, human gingival mesenchymal cells and in-vivo studies on dental pulp cells of rats in which cytotoxicity and cell viability activity were evaluated [4–10]. However, while there are studies on the cellular effects of some commercial preparations [11, 12], there is no study on the changes in cytokine levels in human gingival fibroblasts caused by teething gels with different ingredients.

There is limited research on teething gels. Although most studies are related to the frequency and unconscious use of teething gels, some have clinically evaluated the healing of gingival defects [13–15]. However, there is a gap in the literature regarding the evaluation of the effects on the inflammation process.

Cytokines, produced by epithelial cells, gingival fibroblasts, and immune cells, regulate local symptoms such as gingival inflammation and tissue protection responses [16]. However, there is a lack of studies evaluating the impact of teething gels on the key cytokines that contribute to gingival inflammation.

This study aimed to assess the influence of teething gels on the levels of these cytokines in human gingival fibroblast cells (HGF-1) using in vitro experiments.

## Materials and methods

### Ethics committee approval

Approval for this study was granted by the Afyonkarahisar Health Sciences University Clinical Research Ethics Committee (decision number: 2020/472 dated 06.11.2020). The experimental phase of the study was conducted in the laboratory of the Department of Medical Biochemistry, Faculty of Medicine, Afyonkarahisar Health Sciences University.

### Cell culture preparation

HGF-1, the human primary gingival fibroblast cell line, was obtained from the American Type Culture Collection (ATCC, VA, USA). These cells were cultured in a fully humidified incubator at 37 °C with 5% CO_2_ using Dulbecco’s modified eagle medium (DMEM, Capricorn, Ebsdorfergrund, Germany) supplemented with 10% fetal bovine serum (FBS, Capricorn, Ebsdorfergrund, Germany), 1% penicillin–streptomycin antibiotic solution (Capricorn, Ebsdorfergrund, Germany), and 1% sodium pyruvate (BI Biological Industries, Beit-Haemek, Israel). HGF-1 cells were seeded in 75-cm^2^ culture flasks and grown for 1–2 days before use.

### Preparation of teething gel solutions

Master stock solutions of teething gels were prepared by adding 1 g (g) of each teething gel to 1 milliliter (mL) of medium in Falcon tubes. Master solutions were diluted with medium to prepare ratios of 1:2, 1:4, 1:8, 1:16, 1:64, 1:256, 1:512, and 1:1024. The teething gels used in this study are listed in Table 1. This study comprises seven groups: Control, Lidocaine + Cetylpyridinium Chloride (LH1), Lidocaine + Chamomile Tincture (LH2), 0.5% Hyaluronic Acid (HA1), Hyaluronic Acid + Aloe Vera (HA2), 0.2% Hyaluronic Acid (HA3), and the Choline Salicylate Group (CS).

**Table 1:** The properties, name, contents, manufacturer, and lot number of teething gels

Teething gels property	Teething gels	Contents	Company and lot number
Lidocaine + cetypridinium chloride containing teething gel (LH1)	Calgel^®^	Main contents: Lidocaine hydrochloride, cetylpyridinium chloride Inactive contents: Sorbitol, xylitol, alcohol, sodium saccharin, menthol, sweetener, caramel	GlaxoSmithKline (Brentford, United Kingdom) 21G018
Lidocaine + chamomile tincture containing teething gel (LH2)	Dentinox^®^	Main contents: Lidocaine hydrochloride, hydroxypolyethoxy dodecane, chamomile tincture Inactive contents: Xylitol, sorbitol, propylene glycol, carbomer 934 P, 10% sodium hydroxide solution, polysorbate 20, sodium saccharin, menthol, distilled water	Abdi İbrahim (Istanbul, Turkey) 19L017
Choline salicylate-containing teething gel (CS)	Dencol^®^	Main contents: Choline salicylate Inactive contents: Chlorhexidine gluconate, glycerin, sorbitol, povidone K90, PEG 40 hydrogenated castor oil, citric acid monohydrate and deionized water	Berko ilaç (Istanbul, Turkey) 2,001,209
0.5% hyaluronic acid containing teething gel (HA1)	Aftamed^®^	Main contents: Hyaluronic acid Inactive contents: Water, PEG 400, xylitol, polyvinyl alcohol, carboxymethyl cellulose, PEG 40 hydrogenated castor oil, PVP, PVM/MA copolymer, eicosen copolymer, glyceryl Laurate, polycarbophil sodium saccharinate, monosodium phosphate, flavor, choline alfaskerate, trisodium phosphate dodecahydrate, sodium lactate, disodium EDTA, lactic acid, sodium hydroxide	Bioplax Pharma (Wallington, United Kingdom) 1,902,253,019
Hyaluronic acid + aloe vera containing teething gel (HA2)	Aftadur^®^	Main contents: Hyaluronic acid Inactive contents: Water, polyvinolprolidone (PVP), maltodextrin, propylene glycol, PEG-40 hydrogenated castor oil, xantham extract, potassium sorbate, sodium benzoate, sodium hiluronate, flavor, benzalkonium chloride, disodium EDTA, sodium saccharin, dipotassium glycyrrhizate, aloe vera	Avicenna Farma (Istanbul, Turkey) 0AAL2TU00007
0.2% hyaluronic acid containing teething gel (HA3)	Gengigel^®^	Main contents: Hyaluronic acid Inactive contents: Water, PEG 400, xylitol, polyvinyl alcohol, carboxymethyl cellulose, PEG 40 hydrogenated castor Oil, PVP, PVM/MA copolymer, eicosene copolymer, glyceryl laurate, polycarbophil sodium saccharinate, monosodium phosphate, aroma, choline alfaskerate, trisodium phosphate dodecahydrate, sodium lactate, disodium EDTA, (may contain lactic acid, sodium hydroxide)	Dentocare (London, United Kingdom)

CS, LH1, LH2, HA1, HA2 and HA3, and all prepared dilutions were applied to all groups except the control group.

### MTT assay and calculation of average lethal dose

The number of viable HGF-1 cells after treatment was evaluated using the MTT (3-[4,5-methylthiazol-2-yl]-2,5-diphenyl-tetrazolium bromide) assay. In brief, HGF-1 cells (2 × 10^4^ cells/well) were seeded in a 24-well plate and allowed to attach overnight. The next day, the medium was replaced with fresh medium containing the teething gel solutions and the cells were allowed to grow for 24 h. Then, 100 µl (μL) of MTT (10 mg/mL; Sigma, USA) was added to each well and incubated for 4 h at 37 centigrade (°C); subsequently, the medium was removed and 150 μL of DMSO was added to each well. The plate was shaken for 10 min in the dark at room temperature. The absorbance at 490 nm was read using an ultraviolet/visible spectrophotometer (T70, PG Instruments). The percentage of cell viability was calculated as follows: cell viability (%) = OD_treatment_/OD_control_ × 100 (OD: Optical Density).

The lethal dose 50 (LD50) of the teething gels was established based on the concentrations leading to 50% survival and 50% cell death.

### Determination of total protein and cytokine levels in cell lysates

To determine the total protein and cytokine (Interleukin-1β, Interleukin-6, Interleukin-8, Tumor necrosis factor-α, and Interleukin-10) levels in cells, the culture medium from the flasks containing cells was replaced with LD50 doses of the teething gels. These cells were incubated at 37 °C in a 5% CO_2_ incubator for 24 h.

After incubation, cells from all groups were washed twice with ice-cold phosphate buffered saline (PBS) and then harvested by scraping. The harvested cells were lysed in lysis buffer (100 mM NaH_2_PO_4_, 1% Triton X-100, 1 M HEPES, and 1% protease inhibitor cocktail). The homogenate was centrifuged at 13,000 g for 40 min at 4 °C. The supernatant was collected, and the protein concentration was determined using the bicinchoninic acid (BCA) assay spectrophotometric kit.

**Fig. 1:**
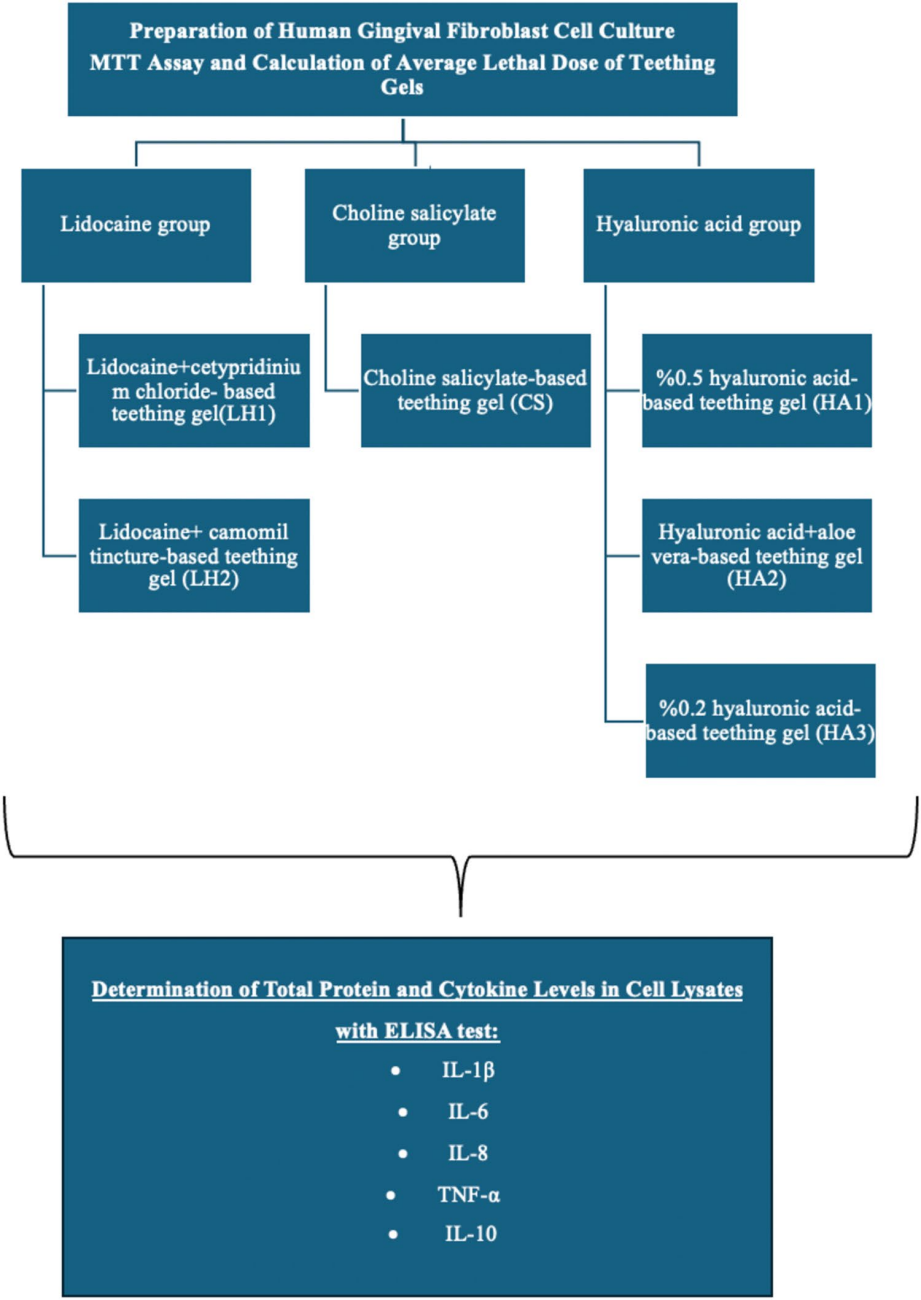
Flowchart of the study. *IL-1β* Interleukin-1β, *IL-6* Interleukin-6, *IL-8* Interleukin-8, *TNF-α* Tumor necrosis factor-α, *IL-10* Interleukin-10

To quantify the cytokines Interleukin-1β (IL-1β), Interleukin-6 (IL-6), Interleukin-8 (IL-8), Tumor necrosis factor-α (TNF-α), and Interleukin-10 (IL-10), human specific ELISA kits (BT Lab, China) were used. The values were expressed as ng (IL-6, IL-8, TNF-α) and pg (IL-1β, IL-10) per mg protein. The flowchart of the study was given in Fig. 1.

### Statistical analysis

This study consisted of 3 biological replicates and 5 method replicates. The data obtained within the study were analyzed using GraphPad Prism version 8.0.1 (GraphPad Software, Inc., CA, USA). The Kolmogorov–Smirnov test was used to assess whether the data followed a normal distribution. As the data exhibited a normal distribution, a one-way analysis of variance (ANOVA) and parametric test was used to compare three or more groups. A post-hoc Dunnett’s correlation analysis and Tukey’s multiple comparison test was conducted to examine the relationships between variables. Statistical significance was considered at a level of p < 0.05.

## Results

The LD50 values, as determined by the MTT test to measure cytokine levels, were 1:128 for the LH1 group, 1:64 for the LH2 group, 1:16 for the CS group, 1:64 for the HA1 group, 1:64 for the HA2 group, and 1:4 for the HA3 group.

A statistically significant reduction in IL-1β levels was observed in HGF-1 cells treated with teething gels in comparison with the control group.

IL-1β levels were significantly lower after treatment with all three ingredients compared with the control group (lidocaine group p = 0.0412; hyaluronic acid group p < 0.0001; choline salicylate group p = 0.0005). IL-1β levels were found to be significantly lower in the hyaluronic acid group than in the lidocaine group (p = 0.0489). No statistically significant difference was found between the lidocaine and choline salicylate groups or the hyaluronic acid and choline salicylate groups in terms of IL-1β (lidocaine–choline salicylate p = 0.1043; hyaluronic acid–choline salicylate p = 0.9837).

IL-6 levels were significantly decreased in HGF-1 cells treated with most teething gels compared with the control group, except in the HA3 group. A statistically significant decrease in IL-6 levels compared with the control group was observed only in the lidocaine-containing group (p = 0.0371). No statistically significant differences were found among the three different teething gel groups (lidocaine–hyaluronic acid p = 0.6822; lidocaine–choline salicylate p = 0.9986; hyaluronic acid–choline salicylate p = 0.7212).

All teething gels led to a statistically significant reduction in IL-8 levels in HGF-1 cells compared with the control group. IL-8 levels were significantly lower for all three treatments compared with the control group (p < 0.0001), but there were no statistically significant differences found between treatments (lidocaine–hyaluronic acid p = 0.3706; lidocaine–choline salicylate p = 0.6297; hyaluronic acid–choline salicylate p = 0.9999).

In most teething gel groups, except HA1, there was a statistically significantly decrease in TNF-α levels in HGF-1 cells compared with the control group (lidocaine p = 0.0002; hyaluronic acid p = 0.0001; choline salicylate p = 0.0008), but there were no statistically significant differences between treatments (lidocaine–hyaluronic acid p = 0.9922; lidocaine–choline salicylate p = 0.9756; hyaluronic acid–choline salicylate p = 0.9112).

IL-10 was found to be significantly decreased in HGF-1 cells treated with teething gels compared with the control group, except for in the HA3 group. The IL-10 levels were significantly lower following all three treatments than in the control group (lidocaine p = 0.0049; hyaluronic acid p = 0.0002; choline salicylate p = 0.0005), but no statistically significant difference was found between the three groups (lidocaine–hyaluronic acid p = 0.8216; lidocaine–choline salicylate p = 0.4139; hyaluronic–choline salicylate p = 0.7647).

**Fig. 2:**
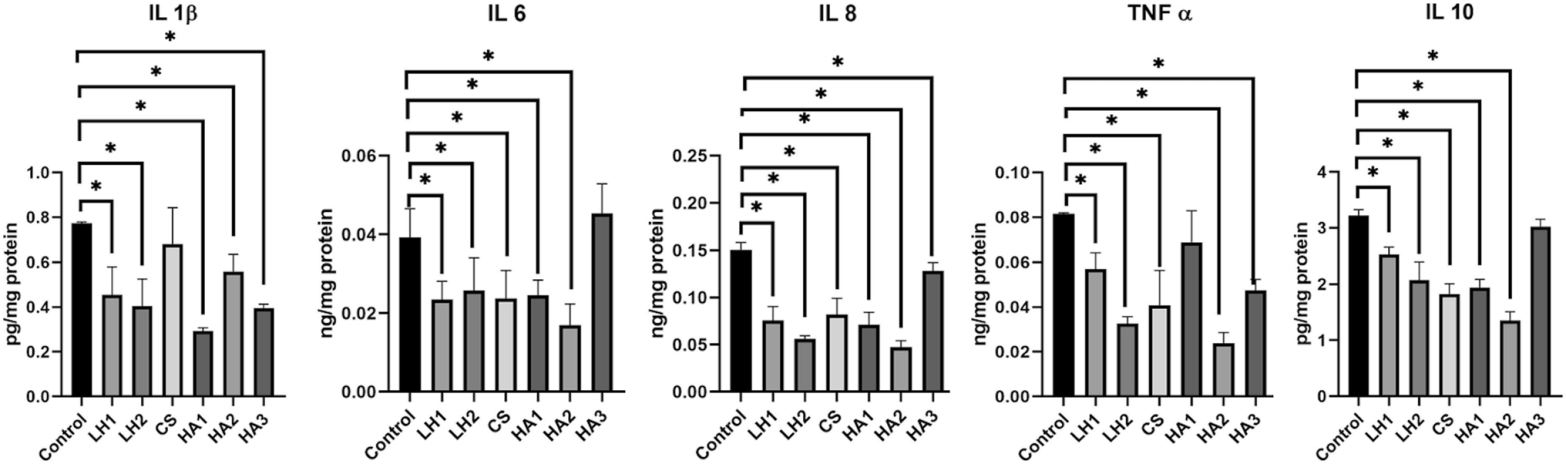
The cytokine levels of HGF-1 applied with teething gels. p < 0.05. In this figure, abbreviated as Lidocaine + cetypridinium chloride containing teething gel (LH1), Lidocaine + chamomile tincture containing teething gel (LH2), Choline salicylate-containing teething gel (CS), 0.5% hyaluronic acid containing teething gel (HA1), Hyaluronic acid + aloe vera containing teething gel (HA2), 0.2% hyaluronic acid containing teething gel (HA3). Interleukin-1β (IL-1β), Interleukin-6 (IL-6), Interleukin-8 (IL-8), Tumor necrosis factor-α (TNF-α), and Interleukin-10 (IL-10). While teething gels are located on the horizontal axis of the graphs, mean and standard deviations of protein weight values of cytokines are located on the vertical axis. One-way Anova,Tukey test

**Fig. 3:**
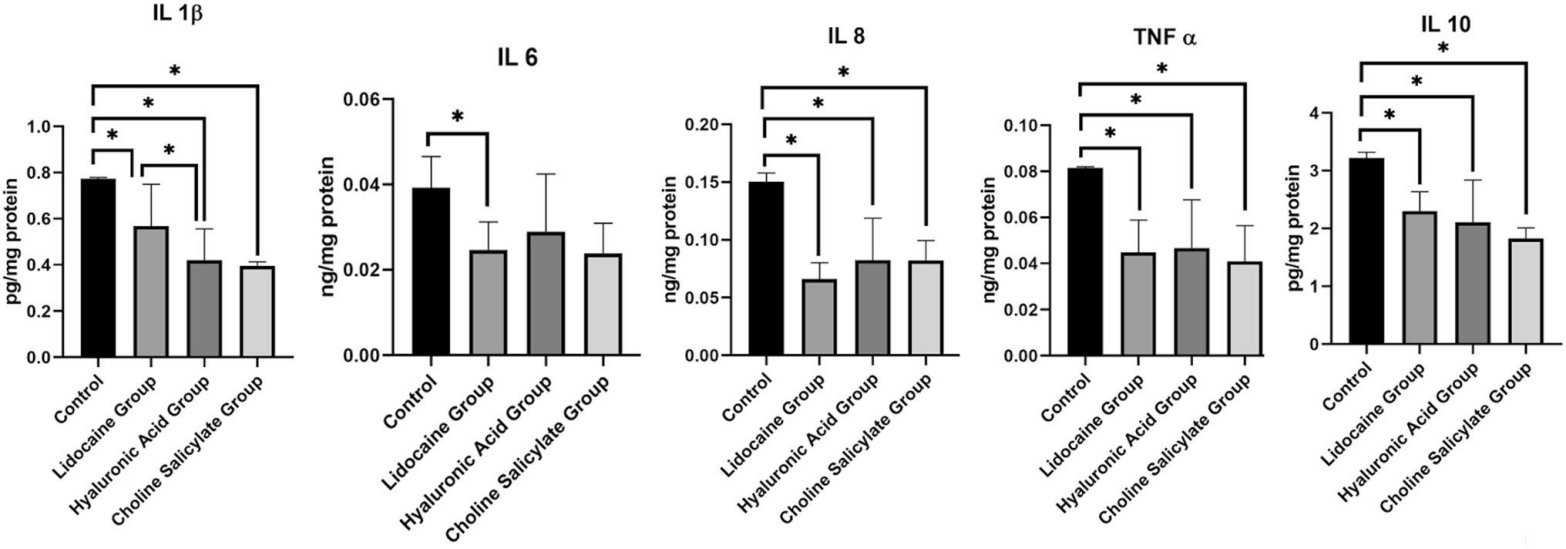
The cytokine levelsof HGF-1cells according to main contents of teething gels. *p < 0.05. While teething gels according to main contents are located on the horizontal axis of the graphs, mean and standard deviations of protein weight values of cytokines are located on the vertical axis. *IL-1β* Interleukin-1β, *IL-6* Interleukin-6, *IL-8* Interleukin-8, *TNF-α* Tumor necrosis factor-α, and *IL-10* Interleukin-10. One-way Anova,Tukey test

The levels of IL-1β, IL-6, IL-8, TNF-α, and IL-10 in the treatment groups are presented in Table 2 and Fig. 2. For a detailed comparison of the cytokine levels based on the teething gel content, refer to Table 3 and Fig. 3.

**Table 2:** Concentrations of Interleukin-1β (IL-1β), Interleukin-6 (IL-6), Interleukin-8 (IL-8), Tumor necrosis factor-α (TNF-α), and Interleukin-10 (IL-10) cytokines in the control groups and teething gel treatment groups

Groups	IL-1β (pg/mg protein)	IL-6 (ng/mg protein)	IL-8 (ng/mg protein)	TNF-α (ng/mg protein)	IL-10 (pg/mg protein)
	Mean + Standard deviations	Mean + Standard deviations	Mean + Standard deviations	Mean + Standard deviations	Mean + Standard deviations
Control	0.774 ± 0.005	0.039 ± 0.007	0.151 ± 0.008	0.082 ± 0.001	3.219 ± 0.107
LH1	0.682 ± 0.161	0.024 ± 0.005^†^	0.076 ± 0.015^†^	0.057 ± 0.007^†^	2.530 ± 0.131^†^
LH2	0.455 ± 0.125^†^	0.026 ± 0.008^†^	0.056 ± 0.003^†^	0.033 ± 0.003^†^	2.073 ± 0.323^†^
CS	0.394 ± 0.018^†^	0.024 ± 0.007^†^	0.082 ± 0.017^†^	0.041 ± 0.016^†^	1.827 ± 0.180^†^
HA1	0.406 ± 0.12^†^	0.025 ± 0.004^†^	0.071 ± 0.013^†^	0.069 ± 0.014	1.940 ± 0.147^†^
HA2	0.559 ± 0.077^†^	0.017 ± 0.005^†^	0.047 ± 0.007^†^	0.024 ± 0.005^†^	1.353 ± 0.154^†^
HA3	0.293 ± 0.014^†^	0.045 ± 0.008	0.128 ± 0.009^†^	0.048 ± 0.005^†^	3.023 ± 0.130

*LH1* Lidocaine + cetypridinium chloride containing teething gel, *LH2* Lidocaine + chamomile tincture containing teething gel, *CS* Choline salicylate-containing teething gel, *HA1* 0.5% hyaluronic acid containing teething gel, *HA2* Hyaluronic acid + aloe vera containing teething gel, *HA3* 0.2% hyaluronic acid containing teething gel

^†^Statistically significant difference between the control group and the indicated group at p < 0.05 by one-way ANOVA and Tukey’s multiple comparison test.

**Table 3:** Comparison of cytokine levels according to teething gel main component

Groups	Control	Lidocaine group	Hyaluronic Acid Group	Cholin Salicylate Group
	Mean + Standard deviations	Mean + Standard deviations	Mean + Standard deviations	Mean + Standard deviations
IL-1β (pg/mg protein)	0.774 ± 0.005^a^	0.568 ± 0.181^b^	0.419 ± 0.136^c^	0.394 ± 0.018^bc^
IL-6 (ng/mg protein)	0.039 ± 0.007^a^	0.025 ± 0.007^b^	0.029 ± 0.014^ab^	0.024 ± 0.007^ab^
IL-8 (ng/mg protein)	0.151 ± 0.007^a^	0.066 ± 0.014^b^	0.082 ± 0.036^b^	0.082 ± 0.017^b^
TNF-α (ng/mg protein)	0.082 ± 0.001^a^	0.045 ± 0.0140^b^	0.047 ± 0.021^b^	0.041 ± 0.016^b^
IL-10 (pg/mg protein)	3.219 ± 0.099^a^	2.302 ± 0.334^b^	2.106 ± 0.734^b^	1.827 ± 0.181^b^

One-way ANOVA, Tukey's multiple comparison test; subscripts ^a–c^ denote no statistically significant difference between column values with the same letter (p > 0.05)

*IL-1β* Interleukin-1β, *IL-6* Interleukin-6, *IL-8* Interleukin-8, *TNF-α* Tumor necrosis factor-α, *IL-10* Interleukin-10

## Discussion

Commercial teething gel preparations have a variety of ingredients, but all are proposed to alleviate local symptoms in the oral mucosa and gingiva through analgesic and anti-inflammatory effects [13, 17]. These local symptoms, including gingival inflammation and physiological responses that safeguard the tissue, are regulated by the pro-inflammatory and anti-inflammatory cytokines synthesized by epithelial cells, gingival fibroblasts, and immune cells infiltrating the gingiva [16]. As far as the authors are aware, no prior study has evaluated the impact of teething gels on the cytokines actively involved in gingival inflammation. Consequently, this study aimed to investigate the cytokine levels of IL-1β, IL-6, IL-8, TNF-α, and IL-10 in human gingival fibroblast cells treated with teething gels in an in vitro setting.

A previous study indicated that IL-1β, an endogenous pyrogenic cytokine, is increased during the tooth eruption process in rats, correlating this phase with an upsurge in IL-1β and an ensuing inflammation process [18]. Furthermore, an increase in IL-1β in the gingival crevicular fluid of adult patients has been associated with gingival inflammation [16]. This study demonstrated that all the tested teething gels, which contain different ingredients, caused a significant decrease in IL-1β levels compared with the control group. In a study investigating lidocaine-containing teething gel (Dynexan Mundgel, Kreussler Pharma, Germany), lidocaine was found to be effective against aphthous ulcers, gingival inflammation, and local symptoms of teething in children between 6 months and 8 years of age when compared with placebo teething gel [17]. The present study employed two different lidocaine-containing teething gels: Lidocaine + chamomile tincture containing teething gel, which led to a greater decrease in IL-1β levels than the control group, and lidocaine + cetypridinium chloride containing teething gel, which yielded results similar to the control group. The differential impact of lidocaine + chamomile tincture containing teething gel, on IL-1β might be attributed to the presence of chamomile, which is known for its anti-inflammatory properties [19].

There is no study in the accessible literature that evaluated the effect of lidocaine + cetypridinium chloride containing teething gel on IL-1β levels. In a randomized controlled clinical trial, which supports the findings of the current study, the effect of lidocaine + cetypridinium chloride containing teething gel and a herbal teething gel (Dantinorm Baby^®^, Boiron, France) on teething symptoms in children was evaluated. Both physicians and parents found the herbal teething gel more successful than lidocaine + cetypridinium chloride containing teething gel in improving symptoms such as inflamed and painful gums, and it was reported that the symptoms lasted longer in the lidocaine group and that undesirable side effects were encountered [20]. In this study, it was determined that teething gels containing hyaluronic acid caused a greater decrease in IL-1β levels compared to teething gels containing lidocaine. In a clinical study evaluating the efficacy of 0.2% hyaluronic acid containing teething gel and lidocaine + cetypridinium chloride containing teething gel in relieving symptoms in infants, it was reported that 0.2% hyaluronic acid containing teething gel reduced symptoms such as pain, swelling, and redness in the gums more rapidly, similar to our findings [13].

In line with present findings, Chen et al. [11] reported significant reductions in IL-1β levels caused by hyaluronic acids with varying molecular weights. In a study conducted on mice, hyaluronic acid caused a decrease in IL-1β levels in experimentally induced inflammation in mouse ear, which have similar characteristics to human gingiva [21]. Furthermore, the application of hyaluronic acid in patients with peri-implantitis led to a significant reduction in IL-1β levels in the gingival crevicular fluid compared with the pre-application period [22]. Hyaluronic acid suppresses inflammation by inhibiting pro-inflammatory molecules without disturbing the internal balance of the prostaglandin pathway. Consequently, hyaluronic acid likely exerts its anti-inflammatory effects by inhibiting Nuclear Factor kappa B (NF-κB) and mitogen-activated protein kinase (MAPK) signaling pathways, thereby suppressing genes related to the inflammatory response, including the IL-1β expression mediated by NF-κB [23].

The choline salicylate-containing teething gel used in current study also elicited a statistically significant reduction in IL-1β levels compared with the control group. Research in various fields has reported that salicylates ingested into the body exert analgesic, anti-pyretic, and anti-inflammatory effects. Additionally, choline salicylate in eye drops decreased levels of pro-inflammatory cytokines such as IL-1β [24]. Although choline salicylate-containing teething gels can be used in the treatment of oral lesions and gingival inflammation owing to tooth eruption, in vivo and in vitro studies are needed to determine the mechanism of action of choline salicylate-containing teething gels on IL-1β levels [25].

IL-6, which plays an important role in mucosal immune responses, is synthesized by endothelial cells, keratinocytes, and fibroblasts. In the presence of gingival inflammation, IL-6 is higher than in healthy tissue, and is a marker of the inflammatory process together with other pro-inflammatory cytokines; moreover the synthesis of IL-6 is increased by IL-1β [16]. The present study revealed that the lidocaine group had significantly lower IL-6 levels than in with the control group. Taniguchi et al. [26] reported that lidocaine treatment in endotoxin-injected rabbits decreased the serum concentration of IL-6. In another in vivo study, it was found that intravenous lidocaine administration caused a decrease in IL-6 levels [27]. Nevertheless, as no research has assessed the impact of lidocaine-containing gels on IL-6 levels in gingival fibroblasts, the study results cannot be directly compared.

In this study, when IL-6 levels were evaluated according to the preparations, it was determined that all teething gels except 0.2% hyaluronic acid containing teething gel caused a statistically significant decrease compared to the control group. In a study, it was reported that hyaluronic acid with different molecular weights could reduce the increased IL-6 level released from inflamed HGF-1 cells, but hyaluronic acid showed similar results to the control group at some molecular weights [11]. In our study where three different teething gels containing hyaluronic acid were used, the variable results of the preparations with the same content may be attributed to their molecular weights or side effects in their content. The lack of sufficient information in the prospectus information provided by the manufacturing companies prevents a definitive judgment from being made with the current results. Further studies are needed on this subject.

A study evaluating the levels of IL-8, which is responsible for immune cell migration and activation in inflamed tissues, reported higher salivary IL-8 levels prior to third molar extraction than post-treatment [28]. In infants, an increase in IL-8 levels has been observed in the gingival sulcus during tooth eruption. However, a significant correlation was found between increased IL-8 levels in the gingival sulcus and gastrointestinal disorders [29]. In the current study, all tested gels led to a statistically significant decrease in IL-8 levels in HGF-1 cells relative to the control group. Among these, the hyaluronic acid + aloe vera containing teething gel exhibited the greatest numerical reduction compared with the control group. In agreement with present findings, Chen et al. [11] examined the efficacy of hyaluronic acid in bacteria-induced gingival inflammation, reporting that hyaluronic acid of varying molecular weights reduced the release of IL-8 from HGF-1 cells. This effect might be attributed to the van der Waals attraction between hyaluronic acid and IL-8, whereby the weakly bonded hyaluronic acid with mucosa-covered IL-8 helps suppress undesired inflammatory responses [30].

Hyaluronic acid + aloe vera containing teething gel, which causes the greatest numerical decrease in IL-8 levels released from HGF-1 cells, contains aloe vera, unlike other hyaluronic acid-containing teething gels. In a study examining the effect of aloe vera on colorectal mucosa in vitro, it was reported that it showed an anti-inflammatory activity by decreasing IL-8 levels [31]. In the current literature reviewed, no study was found evaluating the effectiveness of aloe vera on oral mucosal IL-8 levels. However, it was determined that aloin, a component of aloe vera, showed anti-inflammatory activity and inhibited IL-8 production by epithelial cells in saliva samples of adult patients [32]. The results of this study support that aloe vera may have an effect on IL-8 levels in oral mucosa. However, further studies are needed to reach more definitive conclusions regarding hyaluronic acid + aloe vera containing teething gel and IL-8.

The lidocaine-containing teething gels (LH1, LH2) used in this study also caused a statistically significant decrease in IL-8 levels in HGF-1 cells. When the current literature is examined, studies evaluating the effects of lidocaine on IL-8 levels have been conducted mainly on gastrointestinal cells. In these studies, it has been reported that lidocaine inhibits IL-8 release from intestinal epithelial cells and colon epithelial cells [33–35]. It has been reported that lidocaine may be effective in reducing elevated IL-8 levels and accelerating recovery, especially in patients with ulcerative colitis. In another study, intravenous lidocaine administration was found to be effective in improving intestinal functions that changed after abdominal surgery and in reducing serum IL-8 levels [36]. It is thought that lidocaine, an anesthetic agent, may also be effective in pain sensation by reducing IL-8, which is considered the first inflammatory mediator that induces the sympathetic nervous system in pain sensation [37]. However, since there is no study evaluating the effect of lidocaine-containing teething gels on IL-8 levels in the oral mucosa in the available literature, our findings could not be compared.

Another teething gel used in this study, choline salicylate-containing teething gel, also caused a statistically significant decrease in IL-8 levels compared to the control group. Choline salicylate-containing eye drops have been reported to be used in the treatment of ocular diseases in which pro-inflammatory cytokines such as IL-8 are increased [38–40]. Although it has been reported that choline salicylate-containing teething gels have shown successful results in different oral lesions or teething symptoms and have reduced inflammation and pain, there is no study in the available literature evaluating the effect of choline salicylate on IL-8 levels of HGF-1 cells [25].

TNF-α, one of the main pro-inflammatory cytokines, is an inducer of local inflammatory response during infection. The presence of high serum levels of TNF-α, which can determine the efficiency, duration, and strength of local and systemic inflammatory responses, causes conditions such as tissue damage, catabolic hormone release, and fever [16]. TNF-α levels have been reported to be increased in the gingival crevicular fluid in patients with periodontitis and during orthodontic tooth movement [16, 41]. TNF-α levels in the gingival crevicular fluid of erupting teeth were statistically significantly higher than compared with the period following tooth eruption. Furthermore, TNF-α levels were reported to increase in the gingival crevicular fluid of infants with sleep disorders and fever during tooth eruption [29]. In this study, the evaluation of teething gels based on their content revealed a statistically significant reduction compared with the control group.

The existing body of knowledge lacks studies evaluating the effect of the teething gels used in the current study on TNF-α. Variability has been observed in different in vitro and clinical studies evaluating the efficacy of hyaluronic acid on TNF-α levels [42–44]. Inuyama et al. [42] demonstrated a statistically significant decrease in TNF-α levels following hyaluronic acid application to cultured odontoblast cells (KN-3 cells). Similarly, Lee et al. [43] reported that hyaluronic acid led to a statistically significant reduction in TNF-α levels in lipopolysaccharide-stimulated macrophages compared with the control group. Conversely, a study reported no effect of hyaluronic acid application on TNF-α levels in the gingival crevicular fluid in patients with peri-implantitis [22]. In this study, when considering the three hyaluronic acid-containing teething gels, similar effects were observed in the 0.5% hyaluronic acid containing teething gel and the control group, whereas the other two hyaluronic acid-containing gels resulted in a more pronounced decrease in TNF-α levels. Disparities between the preparations could be attributed to their molecular weights or the auxiliary ingredients.

IL-10, a regulator of inflammatory reactions, is a proliferative and anti-inflammatory cytokine. Concurrently, IL-10 contributes to maintaining bone volume by inhibiting molecules that trigger bone resorption and by regulating bone formation [44]. Although alveolar bone resorption is observed during periodontitis and tooth eruption, bone resorption during tooth eruption is non-pathological. Although bone resorption is essential for the tooth eruption pathway, it decreases in the post-eruption period. A study conducted in mice demonstrated that the highest count of osteoclasts seen in the alveolar bone surrounding the mandibular of the first molar tooth occurred on the third postnatal day, termed major eruption, with a minor eruption on the tenth day. The presence of IL-10 in dental follicles inhibits active bone resorption during tooth eruption, but not on the third and tenth days [45, 46]. However, heightened IL-10 levels resulting from inflammation have been linked to delayed tooth eruption [46]. In the present study, the evaluation of gels by their constituent components revealed that all three types of teething gel exhibited significantly lower IL-10 levels than in the control group.

In this study, topical teething gels other than 0.2% hyaluronic acid containing teething gel applied to HGF-1 cells caused a statistically significant decrease in IL-10 levels. There are limited studies in the available literature evaluating the effectiveness of hyaluronic acid on anti-inflammatory cytokines [11, 43, 47]. In a study consistent with the findings of this study, it was reported that hyaluronic acid inhibited IL-10 production in bacterial inflammation caused by HGF-1 [11]. It was determined that hyaluronic acid with different molecular weights caused a decrease in IL-10 levels in human macrophage cells both in the presence and absence of lipopolysaccharide [43]. It is thought that the molecular weight of the preparation used and the cell type used are effective in the differences between the studies.

Following the comprehensive assessment of the teething gels in this study, it is evident that gels with three different main ingredients caused various suppression of cytokines governing the inflammatory process released from HGF-1 cells. However, despite their efficacy, the potentially cytotoxic effects of teething gels should be considered, particularly given their frequent use in infants, which emphasizes the importance of recommendations from health professionals. When evaluating the teething gels based on their active component, a significant reduction in cytokine levels compared with the control group was found only for lidocaine-containing gels. Given the scarcity of robust clinical data supporting the efficacy of teething gels containing pharmacological ingredients, such as lidocaine, and the substantial number of global case reports detailing severe adverse effects of topical lidocaine gel—especially in young children and infants—the utility and safety of these gels in teething have been scrutinized [3, 48]. Considering the potential side effects, gels containing hyaluronic acid, a natural polysaccharide found in the oral mucosa that suppresses inflammatory cytokines, have a good tolerability profile [14, 49].

Given that the present study was performed using an in vitro experimental model, certain limitations are obvious: a lack of consideration for teething gel removal by saliva in the oral environment, an inability to assess tissue residence time, reliance on a single healthy cell type, and the high technical sensitivity of the tests. Since our study is an in-vitro study, cytokine levels of the cells were measured in separate experimental conditions. Since cytokines affect each other synergistically or antagonistically in cellular activation and cytokines were examined separately in our study, it was not possible to evaluate the clinical efficacy of teething gels. Despite these limitations, this pioneering exploration of the impact of teething gels on cytokines provides a foundation for future investigations in this area.

## Conclusions

In conclusion, this study found that the teething gels containing lidocaine, hyaluronic acid, and choline salicylate suppressed cytokines involved in the inflammatory process to varying degrees. Among the main components, only the lidocaine-containing group significantly reduced all cytokine levels compared to the control group. When assessed as six different commercial formulations, the lidocaine + chamomile tincture, hyaluronic acid + aloe vera, and choline salicylate teething gels significantly decreased all cytokine levels. The fact that preparations with different components exhibited similar effects, or that formulations with the same constituents demonstrated varying effects, may be attributed to the presence of auxiliary active compounds in the products or differences in their molecular weights.

However, more in-vitro and in-vivo studies are needed to compare our findings and to make definite judgements. It is important to conduct prospective, randomized controlled studies in the future, especially to make recommendations for clinical use. Despite all the limitations of the study, the fact that it is the first study on the effect of teething gels on cytokines will be a starting step for further studies on this subject.

## Data Availability

The data that support the findings of this study are available from the corresponding author upon reasonable request. The data are not publicly available due to restrictions, including privacy or ethical considerations.
